# A prospective phase II study of pre-operative chemotherapy then short-course radiotherapy for high risk rectal cancer: COPERNICUS

**DOI:** 10.1038/s41416-018-0209-4

**Published:** 2018-08-17

**Authors:** Simon Gollins, Nicholas West, David Sebag-Montefiore, Shabbir Susnerwala, Stephen Falk, Nick Brown, Mark Saunders, Philip Quirke, Ruby Ray, Philip Parsons, Gareth Griffiths, Tim Maughan, Richard Adams, Chris Hurt

**Affiliations:** 1 North Wales Cancer Treatment Centre, Bodelwyddan, Denbighshire LL18 5UJ UK; 2 Pathology and Tumour Biology, Leeds Institute of Cancer and Pathology, University of Leeds, Leeds, LS9 7TF UK; 3 Leeds Institute of Cancer and Pathology, University of Leeds and Leeds Cancer Centre, Leeds, LS9 7TF UK; 4 Royal Preston Hospital, Fulwood, Preston, PR2 9HT UK; 5 Bristol Haematology and Oncology Centre, University Hospitals Bristol NHS Foundation Trust, Bristol, BS2 8ED UK; 6 Calderdale Royal Hospital, Salterhebble, Halifax, HX3 0PW UK; 7 The Christie NHS Foundation Trust, Withington, Manchester, M20 4BX UK; 8 Centre for Trials Research, Cardiff University, Room 409, Neuadd Meirionnydd, Heath Park, Cardiff, CF14 4YS UK; 9 NCRI RTTQA, Velindre Cancer Centre, Velindre NHS Trust, Velindre Road, Cardiff, CF14 2TL UK; 10 Southampton Clinical Trials Unit, Faculty of Medicine, Univeristy of Southampton, Tremona Road, Southampton, SO16 6YD UK; 11 CRUK/MRC Oxford Institute for Radiation Oncology, University of Oxford, Old Road Campus Research Building, Roosevelt Drive, Oxford, OX3 7DQ UK

**Keywords:** Rectal cancer, Rectal cancer

## Abstract

**Background:**

Neoadjuvant chemotherapy (NAC) allows earlier treatment of rectal cancer micro-metastases but is not standard of care. There are currently no biomarkers predicting long-term progression-free survival (PFS) benefit from NAC.

**Patients and methods:**

In this single arm phase II trial, patients with non-metastatic magnetic resonance imaging (MRI)-defined operable rectal adenocarcinoma at high risk of post-operative metastatic recurrence, received 8 weeks of oxaliplatin/fluorouracil NAC then short-course preoperative radiotherapy (SCPRT) before immediate surgery. Sixteen weeks of post-operative adjuvant chemotherapy (AC) was planned. A pelvic MRI was performed at week 9 immediately post-NAC, before SCPRT. The primary end point was feasibility assessed by completion of protocol treatment up to and including surgery. Secondary endpoints included compliance, toxicity, downstaging efficacy, and PFS.

**Results:**

In total 60 patients were recruited May 2012–June 2014. In total 57 patients completed protocol treatment, meeting the primary endpoint. Compliance with NAC was much better than AC: Comparing NAC vs. AC, the median percentage dose intensity for fluoropyrimidine was 100% vs. 63% and for oxaliplatin 100% vs. 45%. Treatment-related toxicity was acceptable with no treatment-related deaths. Post-NAC MRI showed 44 tumours (73%) were T-downstaged and 22 (37%) had excellent MRI tumour regression grade (mrTRG 1–2). Median follow-up was 27 months with 2-year PFS of 86.2% (10 events). On exploratory analysis, post-NAC mrTRG predicted PFS with no event among those with excellent regression.

**Conclusion:**

The regimen was well tolerated with effective downstaging and encouraging PFS. mrTRG response to NAC may be a new prognostic factor for long-term PFS, but needs validation in larger studies.

## Introduction

Approximately 15,000 patients are diagnosed with rectal cancer annually in the UK and surgery using total mesorectal excision (TME) is the standard of care. Using ‘short-course’ pre-operative radiotherapy (SCPRT) of 25 Gy over 5 working days, followed by TME within a week, reduces pelvic recurrence rates to approximately 5%.^1,2^.

Local recurrence reduction has not impacted on distant metastatic relapse however, which is now the major cause of death. Histopathological features of resected specimens predict increased systemic recurrence risk including >5 mm invasion of disease beyond the muscularis propria (≥T3c),^3,4^ extra-mural venous invasion (EMVI)^5^ and lymph node involvement (LN+).^6^ With such features, distant metastatic relapse is ~6-fold greater than local recurrence.^1,2,7^ MRI scanning is the pre-treatment investigation which can most reliably identify such features.^8–10^

Current UK practice, supported by NICE guidance^11^ is to complete local pelvic treatment with surgery±pre-operative radiotherapy, before considering systemic adjuvant chemotherapy (AC). However, a more recent meta-analysis of four trials incorporating preoperative radiotherapy suggested limited or no benefit for post-operative AC in patients receiving pre-operative (chemo)radiotherapy.^12^ Using NAC as the first received treatment, allows earlier treatment of micrometastases and increased compliance. However, NAC has hitherto not been established as a standard of care in operable rectal cancer.

For tumours staged as operable, i.e., not threatening the surgical circumferential resection margin (CRM) on pre-treatment MRI, no downstaging is required prior to surgery and SCPRT followed by immediate surgery within a week, is a standard approach.^1,2^ This study (COPERNICUS: **C**hem**o**therapy th**e**n **R**adiatio**n** then **I**mmediate **Cu**rative **S**urgery for operable rectal cancer) used 8 weeks of initial NAC with oxaliplatin and 5-Fluorouracil, then SCPRT prior to immediate surgery. It was a UK multicentre, open-label, single arm phase II trial in patients with MRI-staged operable rectal cancer at high risk of developing distant metastases post-operatively.

## Methods

### Eligibility

Eligible patients were adults, ECOG Performance Status 0–1, with histopathologically confirmed rectal adenocarcinoma with the following features:

1. The inferior disease aspect was ≥4 cm from anal verge and the superior aspect was not more superior than the anterior S1/S2 interspace.
2. The mesorectal fascia was not threatened or involved, i.e., tumour was >1 mm from mesorectal fascia on MRI.
3. The primary tumour was mrT3a-b (T3a: tumour invasion ≤1 mm beyond muscularis propria; T3b: invasion >1–5 mm) in the presence of either EMVI or mesorectal lymph nodes(s)/tumour deposit(s) of any size with irregular border or mixed signal intensity. Alternatively the primary tumour was mrT3c (invasion >5–15 mm) or T3d (invasion>15 mm) or T4 (invasion of visceral peritoneum for tumours with a component above peritoneal reflection was permitted although invasion of other organs was not), regardless of EMVI or nodal status. Low tumours did not involve levator ani or anal sphincters.
4. CT scan of chest and abdomen excluded metastatic disease.

Haematological, renal, and hepatic biochemical indices were satisfactory. All patients provided written informed consent to a medical doctor.

### Treatment

#### NAC

Four, 14-day cycles of Oxaliplatin/5-Fluorouracil (OxMdG) were administered via a central venous catheter (day 1 oxaliplatin 85 mg/m^2^ plus levofolinic acid 175 mg over 2 h, then 5-Fluorouracil 400 mg/m^2^ bolus, then 5-Fluorouracil 2400 mg/m^2^ continuous infusion over 46 h).

#### Radiotherapy

Within 14 days following completion of the last 2-weekly cycle of chemotherapy, i.e., within 15–28 days following the first day of the last cycle of chemotherapy, patients commenced treatment with SCPRT. Before commencing SCPRT, haematological and gastrointestinal toxicities should have resolved to NCI CTCAE ≤ grade 1.

COPERNICUS full Radiotherapy Guidelines are included in the Supplementary Material. Briefly, pelvic radiotherapy was planned using oral and intravenous contrast CT simulation with recommended 3 mm CT slices. 25 Gy in 5 daily fractions, prescribed according to recommendations of the International Commission on Radiation Units and Measurements (ICRU-50), was delivered over 5–7 days Monday to Friday as a 3D, conformally planned single-phase treatment, usually with four radiotherapy fields. Gross tumour volume (GTV) was defined using the diagnostic MRI scan and included all macroscopic tumour and any intervening normal rectal wall. Clinical target volume (CTV) was defined in two parts (CTVA and CTVB) and then combined to form the Final CTV (CTVF). CTVA consisted of GTV with a 1 cm margin grown in all directions. CTVB included the mesorectum, the presacral and internal iliac nodal structures. CTVB superior limit was the more superior of 2 cm above the most superior limit of GTV, or the S2/3 interspace. CTVB inferior limit was the more inferior of 2 cm inferior to the most inferior limit of GTV, or the superior limit of puborectalis. The final CTV (CTVF) was derived by combining CTVA and CTVB. The planning target volume (PTV) was derived by adding a 1 cm margin to CTVF in all directions. Verification with cone-beam CT or electronic portal imaging was used on the first 3 fractions of radiotherapy. Radiotherapy quality assurance was carried out via the UK National Cancer Research Institute Radiotherapy Trials Quality Assurance Group (NCRI RTTQA). Pre-accrual quality assurance included completion of two benchmark cases: a delineation exercise and a planning exercise on a pre-contoured patient. These assessments were identical to those which had previously been defined for the ongoing UK phase III ARISTOTLE trial. Gaining previous approval for ARISTOTLE also conferred a centre with COPERNICUS approval. For on-trial, as a minimum, QA consisted of prospective individual case review (both contours and treatment plan) for the first patient from each radiotherapy delivery site. Radiotherapy Protocol compliance of the remaining patients was reviewed using a pre-designed Plan Assessment Form which captured clinically relevant dose–volume metrics.

#### Surgery

Surgery was recommended within 7 days of the last fraction of SCPRT although a gap of 14 days was acceptable.

#### AC

Between 6 and 8 weeks following the date of surgery, patients commenced eight, 14-day cycles of AC using OxMdG, although alternatively a combination of oxaliplatin and capecitabine could be used (a 14-day schedule of oxaliplatin 85 mg/m^2^ IV day one followed by oral capecitabine at 1000 mg/m^2^ twice daily for 9 days).

### Assessments

Toxicity was assessed as per US National Cancer Institute’s Common Terminology Criteria for Adverse Events (CTCAE version 4.03) at the end of each treatment cycle and at the end of SCPRT. Capecitabine compliance was assessed by tablet count at each visit. An MRI was performed at 9 weeks (post NAC, pre-SCPRT) to assess downstaging and tumour regression grade (mrTRG) using a 5-point scale.^13^ Resection specimens were evaluated by local histopathologists as per detailed trial-specific guidelines (see appendix E of the trial protocol, included as Supplementary Material). The 5-point Dworak system was used to assign a pathological tumour regression grade (pTRG), mirroring mrTRG.^14^ Sites sent formalin-fixed, paraffin-embedded blocks of pre-treatment and surgical resection material to Leeds Institute of Cancer and Pathology, University of Leeds. All glass haematoxylin and eosin stained slides were also sent from the biopsy and resection specimens for scanning to create a permanent record (http://www.virtualpathology.leeds.ac.uk/clinical/colorectal/copernicus) and facilitate central review of pTRG and tumour cell density (TCD) calculation.^15^

TNM Classification of Malignant Tumours 5th edition was used for histopathological staging. Histopathological substages T3a-d were defined similarly to those used for MRI. Pathological complete response (ypT0ypN0) was confirmed as follows: Where tumour cells could not be found on the first assessment of up to five blocks of tumour, the whole area of the tumour/fibrotic scar was embedded and examined. If no tumour cells could be seen following assessment of these extra blocks, then three deeper levels were taken and examined from each tumour/fibrotic scar block. ypT0ypN0 was confirmed if no tumour cells were identified. There were no ypT0N+cases.

TCD^15^ was measured by digitally scanning the glass haemotoxylin and eosin-stained slides at ×200 magnification and annotating around the tumour. Approximately 300 random points within this area were then manually assessed to determine the underlying tissue components and calculate the TCD. For resected tumour TCD was expressed as either the ‘whole tumour’ TCD when the outlining included scar, or alternatively the area of the greatest TCD when annotating a 3 × 3 mm^2^ area in the region of apparent greatest residual tumour.

Post-operative morbidity was assessed at 30 days post-surgery. Patients were followed up to assess disease status at 6 months and 12 months following surgery, with a CT scan mandated at 12 months. Investigations and follow-up beyond 12 months were done as per institutional standard.

### Statistics

This was a single arm phase II trial. The primary endpoint was completion of protocol treatment up to and including surgery defined as starting NAC, and completing SCPRT and surgery. If >92% (as was found in CR07,^2^ and not <80%, of patients completed surgery then it would warrant further investigation in the Phase III setting. Based on an A’Hern design^16^ looking at proportion of patients completing surgery and setting p0 = 0.80, p1 = 0.92, 90% power, alpha = 0.1, 57 patients were required (with 50 patients completing surgery counting as success).

Secondary endpoints included compliance, toxicity, histopathological and radiological assessment of downstaging efficacy, and PFS. Exploratory analyses investigated the relationship between measures of downstaging efficacy and PFS.

Data were analysed according to a pre-specified analysis plan using the Stata SE 14 statistical package. All analyses were by intention to treat except toxicity analyses, which were conducted only in those patients who had some treatment during the related treatment phase, and the surgical complications analysis only in those who had surgery. Clopper-Pearson exact binomial method was used to calculate confidence intervals for the primary endpoint. We calculated % of total dose (actual total dose divided by protocol total dose) and % dose intensity (actual dose intensity [dose per unit time] divided by protocol dose intensity) for each protocol drug as measures of compliance. Baseline and pre-surgical weight was compared using a Wilcoxon signed rank test. Univariable ordinal regression was used to look for associations between categorical (number of baseline MRI risk factors (out of T≥3c or N1-2 or EMVI+), mrTRG, pTRG,) and continuous variables (TCD). We calculated progression free survival (PFS) from date of registration to when a failure event (death, or confirmed evidence of distant metastases or loco-regional progression) occurred. Patients who were event-free were censored at the time they were last known to be event free. Follow-up time distribution was estimated using the reverse Kaplan–Meier method^17^ with patients censored at date of death or last trial assessment. We estimated event time distributions with the Kaplan–Meier method and looked for relationships between PFS and potential predictors using Cox regression in univariable models.

## Results

**Fig. 1:**
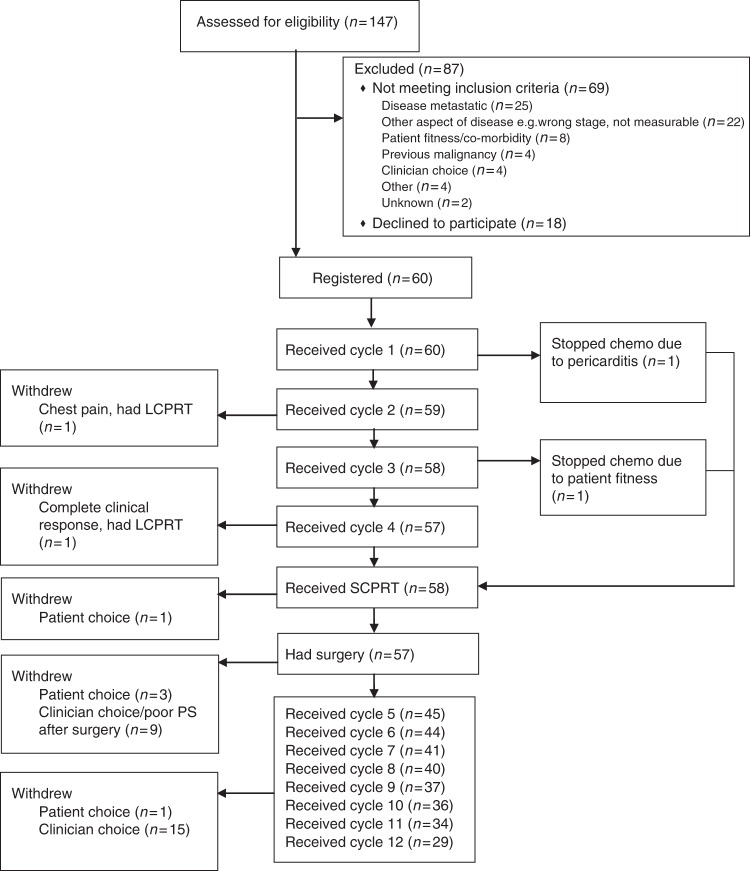
Participant flow diagram

In total 60 patients were enroled from 14 UK centres between 25 May 2012 and 11 June 2014 (Fig. 1). At the time of analysis, all patients had completed at least 1 year of post-surgical follow-up or withdrawn from the study. Patient and tumour baseline characteristics are shown in Table 1. Pre-treatment MRI showed that 35 (58%), 14 (23%) and 11 (18%) patients had 1, 2 or 3 high-risk features, respectively.

**Table 1:** Baseline characteristics and 9 week MRI results

	Baseline	9 weeks
	*n* (%)^a^	*n* (%)^a^
Patients enroled		
* N*	60	
Age (years)		
Median (IQR, range, *n*)	63 (56.5–70, 38–79, 60)	
Gender		
Male	44 (73.3)	
Female	16 (26.7)	
ECOG performance status		
0	55 (91.7)	
1	5 (8.3)	
Predominant differentiation of primary tumour		
Well	5 (8.3)	
Moderate	49 (81.7)	
Poor	2 (3.3)	
Unknown	4 (6.7)	
Time from MRI scan to registration (weeks)		
Median (IQR, range, *n*)	3.7 (2.6–4.6, 0–5.9, 60) before reg	9.4 (8.7–10,7.4–12.4,58) after reg
MRI–Craniocaudal length (mm)		
Mean (SD, range, *n*)	49 (11.9, 28–80, 58)	34.3 (13.5, 7–70, 52)
MRI–Height from anal verge (mm)		
Mean (SD, range, *n*)	79 (21.5, 40–140, 58)	82.9 (21.8, 35–140, 52)
MRI T Stage		
T0		4 (7)
T1		1 (2)
T2	1 (1.7)	28 (47)
T3a	17 (28.3)	6 (10)
T3b	24 (40)	13 (22)
T3c	14 (23.3)	4 (7)
T3d	1 (1.7)	0 (0)
T4	3 (5)	0 (0)
Missing	0 (0)	4 (7)
MRI N Stage		
N0	7 (11.7)	38 (63)
N1	39 (65)	18 (30)
N2	14 (23.3)	2 (3)
Missing	0 (0)	2 (3)
MRI M Stage		
M0	60 (100)	58 (97)
Missing	0 (0)	2 (3)
MRI–CRM involvement		
Clear (>1 mm)	59 (98.3)	57 (95)
Missing data	1 (1.7)	2 (3)
MRI–Extramural venous invasion		
Positive	25 (41.7)	9 (15)
Negative	35 (58.3)	49 (82)
Missing	0 (0)	2 (3)
Number of baseline MRI risk factors out of T≥3c or N1-2 or EMVI+		
3	11 (18.3)	
2	14 (23.3)	
1	35 (58.3)	
MRI tumour regression grade		
Complete regression (TRG1)		6 (10)
Good regression (TRG2)		16 (27)
Moderate regression (TRG3)		15 (25)
Slight regression (TRG4)		12 (20)
No regression (TRG5)		7 (12)
Missing data		4 (7)

^a^Unless otherwise indicated, denominator is 60

### Toxicities

Toxicities during neoadjuvant (end of cycle 1 to post SCPRT assessment) and adjuvant (end of cycle 5 to end of cycle 12) treatment periods are shown in Table 2. The rate of any grade 3 + toxicity was similar in each treatment period: neoadjuvant 24/60 (40%) and adjuvant 15/45 (33%), with neutrophil count decrease being the most common toxicity in each case: 12/60 (20%) and 5/45 (11%), respectively. There were no deaths during treatment (including the post-surgical period). There was no evidence of a difference between baseline (median: 84.1 kg, IQR: 78.1–92.1) and pre-surgical (median: 85.3 kg, IQR: 76.1–92.7) weight (*z* = 0.796, *p* = 0.426, *n* = 56). Post-surgical complications within 30 days are shown in Supplementary Online Table 1 including three patients (5%) who suffered an anastomotic dehiscence. Out of the 57 patients who had surgery, 49 (86%) were discharged within 30 days, a median of 7 (IQR: 6–11) days after surgery.

**Table 2:** CTCAE v4.02 grade 3+ toxicity in patients during neoadjuvant (week 1 to post SCPRT assessment) and adjuvant (post cycle 5 to post cycle 12 assessment) treatment

System organ class	Adverse event	Neoadjuvant *n* (%)^a^	Adjuvant *n* (%)^b^
Any	Any	24 (40)	15 (33)
Cardiac disorders	Pericarditis	1 (2)	0 (0)
Gastrointestinal	Abdominal pain	0 (0)	2 (4)
	Diarrhoea	1 (2)	3 (7)
	Dyspepsia	1 (2)	0 (0)
	Gastritis	1 (2)	0 (0)
	Nausea	1 (2)	1 (2)
	Rectal obstruction	0 (0)	1 (2)
	Stomatitis	2 (3)	0 (0)
General disorders and administration site conditions	Injection site reaction	0 (0)	1 (2)
	Localised oedema	1 (2)	0 (0)
Infections and infestations	Upper respiratory infection	1 (2)	2 (4)
Injury, poisoning and procedural complications	Vascular access complication	1 (2)	0 (0)
Investigations	Neutrophil count decreased	12 (20)	5 (11)
	Platelet count decreased	2 (3)	1 (2)
	Weight loss	0 (0)	1 (2)
	White blood cell decreased	1 (2)	1 (2)
Metabolism and nutrition disorders	Hyperuricemia	0 (0)	1 (2)
Nervous system disorders	Neuropathy	1 (2)	0 (0)
	Syncope	1 (2)	0 (0)
Psychiatric disorders	Agitation	1 (2)	0 (0)
	Anxiety	1 (2)	0 (0)
Respiratory, thoracic and mediastinal disorders	Cough	1 (2)	0 (0)
Skin and subcutaneous tissue disorders	Palmar-plantar erythrodysesthesia syndrome	0 (0)	1 (2)
	Rash	1 (2)	0 (0)
Vascular disorders	Thromboembolic event	2 (3)	0 (0)

^a^denominator *N* = 60 (all patients starting neoadjuvant chemotherapy)

^b^denominator *N* = 45 (all patients starting adjuvant chemotherapy)

### Compliance

**Fig. 2:**
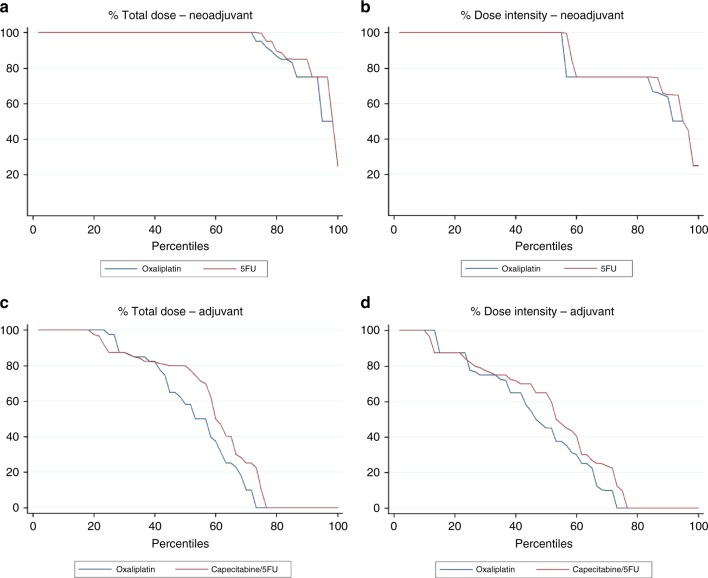
Total dose and dose intensity curves for neoadjuvant and adjuvant chemotherapy

Compliance with NAC was much better than AC, as shown in Fig. 2. In total 45/60 (75%) of patients started AC. 22/60 (37%) of patients switched from 5FU to capecitabine during AC. The median percentage total dose and dose intensity of 5FU/capecitabine was 100 (IQR: 97–100) and 100 (IQR: 75–100) respectively during NAC and 80 (IQR: 5–88) and 63 (IQR: 5–81) respectively during AC. The median percentage total dose and dose intensity of oxaliplatin was 100 (IQR: 93–100) and 100 (IQR: 75–100) respectively during NAC and 58 (IQR: 0–98) and 45 (IQR: 0–77) respectively during AC.

Of the 58 patients who started SCPRT (see Fig. 1), the median time from the start of last cycle of NAC to the start of SCPRT was 24 days (IQR: 19–27, Range: 13–103), all patients received SCPRT at full protocol dose, and only 1 patient experienced a non-logistical delay during SCPRT (due to rheumatoid arthritis flare-up).

In total 57/60 (95%, 90% CIs: 88–99) patients had surgery, the majority (43, 75%) had an anterior resection, 3 (5%) a Hartmann’s resection and 11 (19%) an abdominoperineal excision. The median time from finishing pre-surgical treatment to having surgery was 7 days (IQR: 4–12, Range: 1–34, *n* = 57). The median time from surgery to start of adjuvant chemotherapy was 63 days (IQR: 53–79, Range: 40–133, *n* = 45). Three patients did not have surgery: one patient refused surgery because of anxiety with no recurrence at 18 months post-registration. Contrary to protocol, two patients received long-course chemoradiation rather than SCPRT. One of these later underwent a successful resection and in the other a ‘watch and wait’ approach was adopted with no recurrence at 17 months post-registration.

All 9 radiotherapy centres passed pre-accrual QA prior to entering patients into the trial. Of the 58 patients having SCPRT, the first patient’s contours and plans from each radiotherapy centre underwent prospective review prior to radiotherapy starting. Only one centre was required to undergo repeat prospective review because they submitted their first patient’s data for review after the patient had started their RT. One additional centre was requested to make some minor contouring amendments prior to RT—this was completed successfully and a further patient review was not necessary. The remaining 48 plans were assessed using the Plan Assessment Form. All plans were considered acceptable.

### Efficacy

#### Radiological downstaging

Table 1 shows the results from the post-NAC week 9 MRI compared to baseline MRI. T Stage was downstaged in 44/60 (73%), unchanged in 12/60 (20%), and missing for 4/60 (7%) of patients. N Stage was downstaged in 36/60 (60%), unchanged in 22/60 (37%), and missing for 2/60 (3%) of patients. There was no recorded progression in either T or N stage during NAC. 22 tumours (37%) showed excellent regression (mrTRG 1-2). EMVI reduced from 25/60 (42%) pre-operatively to 9/60 (15%) on the week 9 MRI.

#### Pathological downstaging

Surgical procedure information and pathology results are shown in Table 3. In total 7/57 (12%) patients were found to have had a complete pathological response, confirmed by a central review that included a check of the number of deeper levels cut on the entirely embedded scars. Compared to baseline MRI, histopathological (ypT) T stage was downstaged in 42/57 (74%), unchanged in 11/57 (19%) and upstaged in 4/57 (7%) of patients. Histopathological (ypN) N stage was downstaged in 37/57 (65%), unchanged in 16/57 (28%), and upstaged in 4/57 (7%) of patients. The incidence of histopathological EMVI + in the resected specimen (11/57: 19%) was similar to that shown on the week 9 MRI (9/58: 16%, Table 3).

**Table 3:** Surgery and pathology

	*n* (%)^a^
Surgical procedure	
Abdominoperineal excision	11 (19)
Anterior resection	42 (74)
Hartmann’s	3 (5)
Anterior resection & prophylatic TAH & BSO	1 (2)
Does the patient have a defunctioning stoma	
Yes	43 (75)
No	11 (19)
Missing	3 (5)
If yes, what type?^b^	
Ileostomy	32 (74)
Colostomy	10 (23)
Missing	1 (2)
If yes, intention?^b^	
Temporary	29 (67)
Permanent	14 (33)
Post-operative pathology	
pT0	7 (12)
pT1	3 (5)
pT2	19 (33)
pT3a	9 (16)
pT3b	10 (18)
pT3c	8 (14)
pT4a	1 (2)
pN0	39 (68)
pN1	13 (23)
pN2	5 (9)
R0 (>1 mm from margin)	56 (98)
R1 (≤1 mm from margin)	1 (2)
Number of lymph nodes examined	
Median (IQR, range, *n*)	19 (14–25, 1–48, 57)
Number of lymph nodes positive	
0	39 (68)
1–2	11 (19)
3–5	6 (11)
13	1 (2)
Extramural venous invasion	
Yes	11 (19)
No	46 (81)
Distance to CRM (mm)	
Median (IQR, range, *n*)	12 (6–15.5, 1–50, 47)
pTRG	
No regression	7 (12)
Minimal regression	17 (30)
Moderate regression	14 (25)
Good regression	12 (21)
Complete regression	7 (12)
Plane of resection of mesorectum	
Muscularis propria	2 (4)
Intramesorectal	6 (11)
Mesorectal	42 (74)
Missing	7 (12)
Plane of abdomnio-perineal excision^c^	
Levator	3 (27)
Sphincteric	5 (46)
Intrasphincteric/perforated	0
Missing	3 (27)
Distance of tumour to distal surgical margin (mm)	
Median (IQR, range, *n*)	30 (20–50, 5–100, 48)
Involvement of distal margin?	
No	54 (95)
Missing	3 (5)
Distance of tumour to proximal surgical margin (mm)	
Median (IQR, range, *n*)	182.5 (120–255, 50–460, 46)
Involvement of proximal margin?	
No	53 (93)
Missing	4 (7)
Is there any evidence of tumour perforation?	
Yes	2 (4)
No	53 (93)
Unknown	2 (4)
Peritoneal involvement? (ypT4b disease)	
Yes	1 (2)
No	50 (88)
Missing	6 (11)
Biopsy TCD	
Median (IQR, range, *n*)	37.2 (22.7–44.2, 6.3–58.5, 59)
Resection greatest TCD	
Median (IQR, range, *n*)	21.4 (2.7–39.1, 0–59.9, 57)
Resection whole TCD	
Median (IQR, range, *n*)	8.7 (1.3–16.1, 0–38.2, 57)
Resection whole TCD as % of biopsy TCD	
Median (IQR, range, *n*)	19.4 (3.2–53.8, 0–468.9, 57)

^a^unless otherwise indicated, denominator *N* = 57

^b^denominator *N* = 43, i.e., those with defunctioning stoma

^c^denominator *N* = 11, i.e., those who had abdominoperineal excision

Likewise waterfall plots of TCD showed a marked response (Supplementary Online Fig. 1). Median biopsy TCD was 37.2%, reduced to 8.7% in resection whole TCD.

#### Relationship between mrTRG and resection pathology

We found a significant association between mrTRG and pTRG (*χ*^2^ = 26.5, *p* = 0.048), resection greatest TCD (ordinal regression *z* =−3.77, *p* < 0.001), and resection whole TCD (*z*=−3.77, *p*<0.001) in the 54 patients with non-missing data.

#### Relationship between pTRG and TCD

Univariate ordinal regression showed no evidence of a relationship between pTRG and the number of baseline MRI risk factors (*z*=−0.06, *p* = 0.951) or biopsy TCD (*z* = 0.29, *p* = 0.774, *n* = 57), but strong relationships between pTRG and resection greatest TCD (*z*=−5.70, *p* < 0.001, *n* = 57), resection whole TCD (z =−5.41, *p* < 0.001, *n* = 57), and resection whole TCD as a percentage of biopsy TCD (*z*=−4.03, *p* < 0.001, *n* = 57).

#### PFS

The median follow-up for PFS was 27.3 months (IQR: 22.3–31.1). Of the 60 patients enroled in the trial, 10 had progression (8 distant (6 lung, 1 liver, 1 small bowel), and 2 local recurrences) at the time of analysis. The PFS rate at 2 years was 86.2% (95% CIs: 74.3–92.9). One patient died after 2.3 years.

#### Relationship between measures of downstaging and PFS

**Fig. 3:**
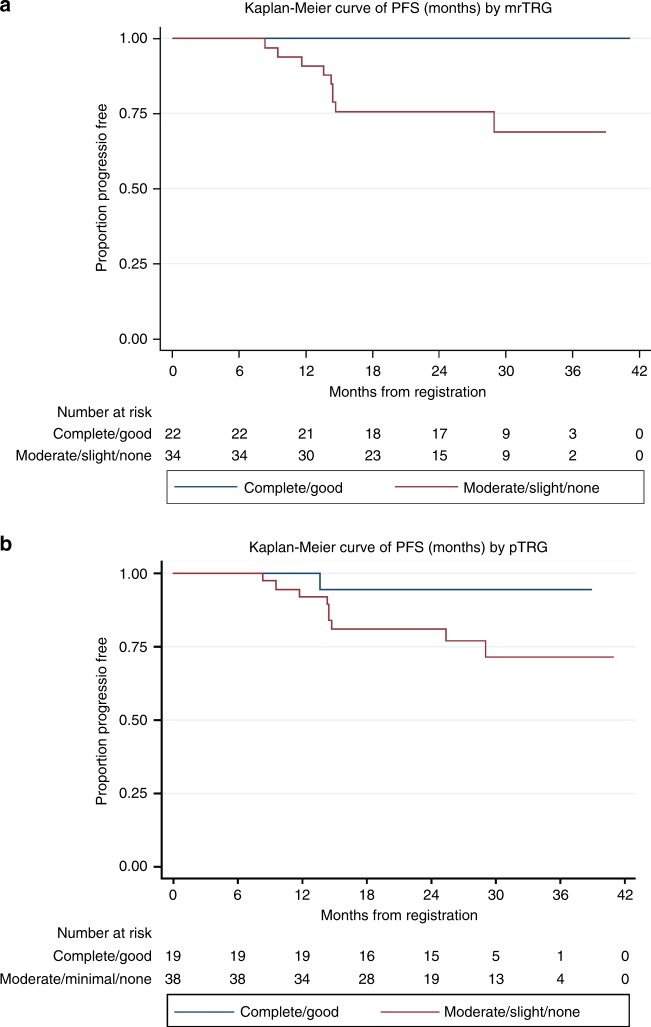
**a** Kaplan–Meier curve of PFS (months) by mrTRG

Strikingly, none of the 22 patients with an excellent response to NAC on the week 9 MRI (mrTRG 1–2) had a progression event. All 10 progression events occurred in the 34 patients with mrTRG 3–5 (Fig. 3a).

Univariate cox regression showed a weak association between pTRG (when split by complete/good vs moderate/minimal/none) and PFS (see Fig. 3b): HR = 4.76 (95% CIs: 0.60–37.61, *p* = 0.139). When TCD was included as a continuous variable in a univariate cox regression, there was some evidence that resection greatest TCD was associated with worse PFS (HR 1.03, 95% CI: 1.00–1.07, *p* = 0.066) but evidence for association with other TCDs was weak: biopsy TCD HR 1.01 (95% CI: 0.96–1.06, *p* = 0.725), resection whole TCD HR 1.04 (95% CI: 0.99–1.10, *p* = 0.137).

## Discussion

This study has demonstrated the safety and feasibility of introducing NAC prior to SCPRT and surgery. There was a strikingly better compliance to NAC versus AC, rates of Grade 3+ toxicity were acceptable (≤40%) and post-operative complications were similar to what might be expected with SCPRT alone followed by immediate surgery.^2^

In keeping with our findings a randomised phase II study (GCR3) of neoadjuvant versus post-operative adjuvant oxaliplatin and capecitabine in 108 patients treated with chemoradiation and surgery, demonstrated less toxicity (*p* = 0.0004) and better compliance (*p* < 0.0001) for NAC compared with AC.^18^

In attempting to successfully treat micro-metastases, which are the main cause of rectal cancer death, there is thus a strong rationale for assessing initial NAC in trials powered for 3-year PFS. A further potential benefit of NAC is earlier closure of temporary stomas. The quality of surgery in the current study was good, with 74% of specimens resected in the mesorectal plane, suggesting that NAC then SCPRT is safe and not compromising surgical quality. The overall PFS rate at 2 years of 86.2% in this MRI-selected high risk group of patients is encouraging.

Only one previous observational study examined NAC then SCPRT then surgery, recruiting 67 patients with cT3-4 tumours from 1997 to 2008.^19^ Patients received eight weeks of NAC (5-FU-based for the first 28 patients then oxaliplatin-5FU for the subsequent 39), followed by SCPRT (20 Gy in five fractions) then surgery within the following week. Although histopathological downstaging was seen in 51% of patients, no pCR was reported.

In contrast the current regimen suggested greater anti-tumour activity, with superior T-downstaging and 7 tumours (12%) showing a ypT0ypN0 pCR. Reasons for this discrepancy may include the higher radiotherapy dose used in the current study (25 vs. 20 Gy), the use of both oxaliplatin and 5FU for all patients, and MRI selection (no MRI staging was used in Ciammella et al.^19^).

The Dutch TME trial failed to demonstrate any histopathological T stage downstaging with SCPRT alone of 5 × 5 Gy followed by surgery within a week.^20^ However very significant downstaging was seen in the current study, so NAC appears of importance for the observed anti-tumour activity. The week 9 MRI demonstrated conclusively that OxMdG NAC alone produced marked tumour downstaging.

The relative contributions of NAC and SCPRT to histopathological downstaging remain unclear and are potentially complex. Theoretical advantages to NAC before radiotherapy include improved tumour response through downstaging and increased oxygenation/radiosensitisation. Theoretical disadvantages include delayed radiotherapy and selection of radioresistant clones.^21^ There are currently no phase III studies examining NAC then SCPRT. The use of SCPRT for rectal cancer is decreasing in the UK. However, this trend could change dependent on the results of the phase III RAPIDO trial (NCT01558921), which completed accrual of 920 patients in June 2016. This randomised patients with locally advanced rectal cancer to standard chemoradiation then surgery, versus an experimental arm of SCPRT followed by NAC then surgery.

A meta-analysis has highlighted limited efficacy of AC following preoperative radiotherapy.^12^ Several factors potentially reduce the effectiveness of AC in rectal cancer. Morbidity from surgery and radiotherapy can delay initiation and reduces tolerance of AC. In addition, the presence of post-operative stomas, expected in approximately 80% of patients,^22^ can impair or stop chemotherapy delivery altogether because of diarrhoea.^23^ The high response rate to initial NAC in the current study suggests that future comparison of pre- versus post-operative chemotherapy may be justified.

The UK has a high standard of routine pelvic MRI reporting following UK-wide initiatives such as the LOREC educational programme.^24^ MRI-defined TRG has been suggested to predict survival outcome post long-course chemoradiation^13^ and is increasingly reported in routine clinical practice following pre-operative chemoradiation. Theoretically the baseline MRI scan could over-stage some patients in terms of their high-risk factors, leading to over-treatment. The loss of high-risk histopathological features in the eventually-resected specimen, due to the downstaging effect of neoadjuvant chemotherapy, makes if difficult to quantify this risk, although because of previously-reported strong correlation of MRI and histopathological high-risk features, we consider this risk low.^8–10^

Response to NAC assessed by MRI by RECIST criteria, was described in the EXPERT trials, which used 12 weeks oxaliplatin/capecitabine before chemoradiotherapy and then surgery in 186 subjects. The radiological response rate was 63%, and only 2 patients (1%) progressed,^25–27^ echoing current findings. Pathological TRG following chemoradiation and surgery has recently been suggested to be both a prognostic factor and an individual patient level surrogate for disease-free survival within the randomised CAO/ARO/AIO-04 trial.^28^

To our knowledge mrTRG has not been described in rectal cancer post-NAC alone, as in the current study. We found that mrTRG was well correlated with pTRG and resection TCD but better than them at predicting PFS, although a hazard ratio could not be calculated due to no events in the 22 excellent responders. The use of week 9 MRI post-NAC but before SCPRT most directly assessed the effect of NAC, without the addition of radiotherapy, which potentially affected subsequent pTRG and resection TCD. However, because of an overall limited number of events, it was not possible to perform multivariable regression to assess whether or not mrTRG remained a strong independent predictive marker for PFS, nor was it possible to assess whether or not it significantly outperforms other potential predictors such as pathological response.

Histopathological TCD provides a novel continuous measure of tumour regression, as opposed to the categorical pTRG measures, such as Dworak. Hitherto, TCD has not been reported in assessing rectal cancer response but here clearly demonstrated marked response, which correlated strongly with Dworak pTRG. Possibly relevant to future studies, the area of greatest residual TCD appeared to be more strongly associated with PFS than both whole tumour TCD (including scar) and pTRG. However, statistical power was limited due to the limited patient numbers and events.

In conclusion, at present there are no established biomarkers in patients receiving NAC that can predict those patients who may demonstrate a PFS benefit compared to those who will not. Our findings suggest that mrTRG following NAC may be a prognostic factor for PFS and are hypothesis-generating but need validation in future, larger studies. If confirmed, mrTRG has potential for use as a short-term surrogate in future studies of intensified NAC treatment strategies aimed at improving PFS in this high-risk group of patients.

### Availability of data and material

The trial was sponsored by Cardiff University. Central trial coordination was by the Centre for Trials Research, Cardiff, including data collection and statistical analyses, where data supporting the results reported in the article can be found.

## Electronic supplementary material

Supplementary Figure 1

Supplementary Table 1
