# Supplementary material for: A prospective phase II study of pre-operative chemotherapy then short-course radiotherapy for high risk rectal cancer: COPERNICUS
Source: Br J Cancer. 2018 Aug 17;119(6):697–706. doi: 10.1038/s41416-018-0209-4 (PMC6173784; doi:10.1038/s41416-018-0209-4)
Supplement: Supplementary file 1 — Supplementary Figure 1 [file 41416_2018_209_MOESM1_ESM.docx]

**Supplementary Online Figure 1. Waterfall plots of TCD by pTRG**

| 1. **Biopsy TCD (p=0.774)**   **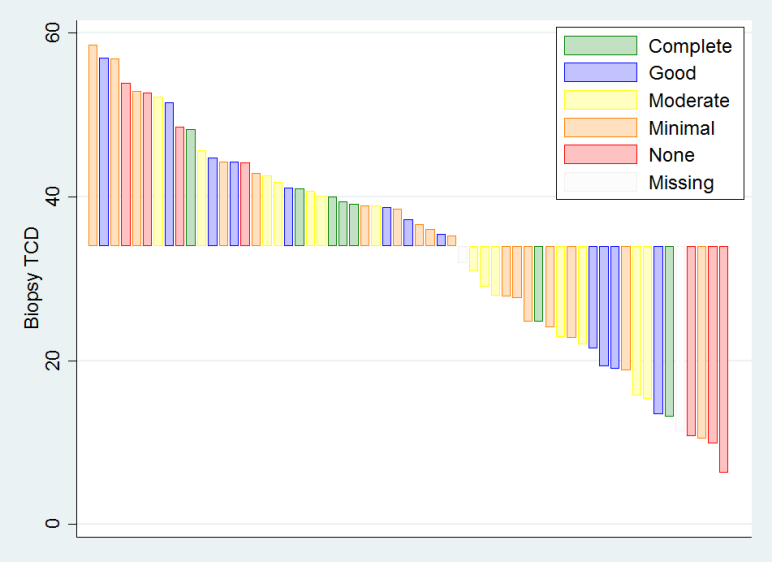** | 1. **Resection greatest TCD (p<0.001)**   **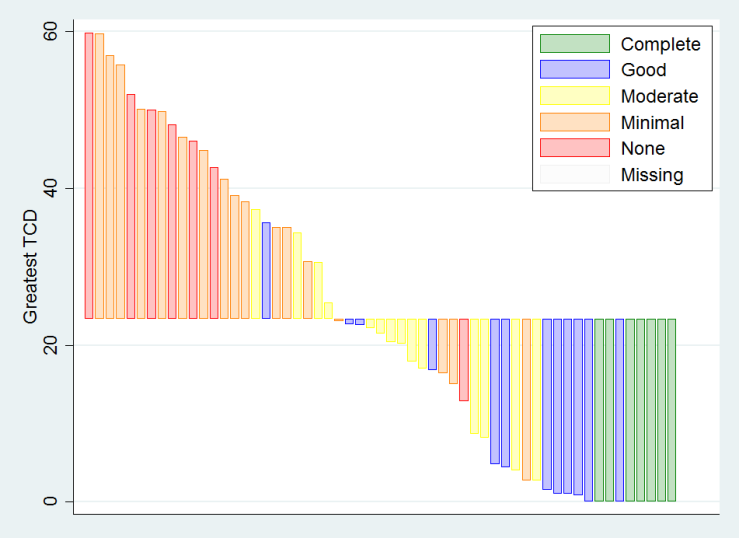** |
| --- | --- |
| 1. **Resection whole TCD (p<0.001)**   **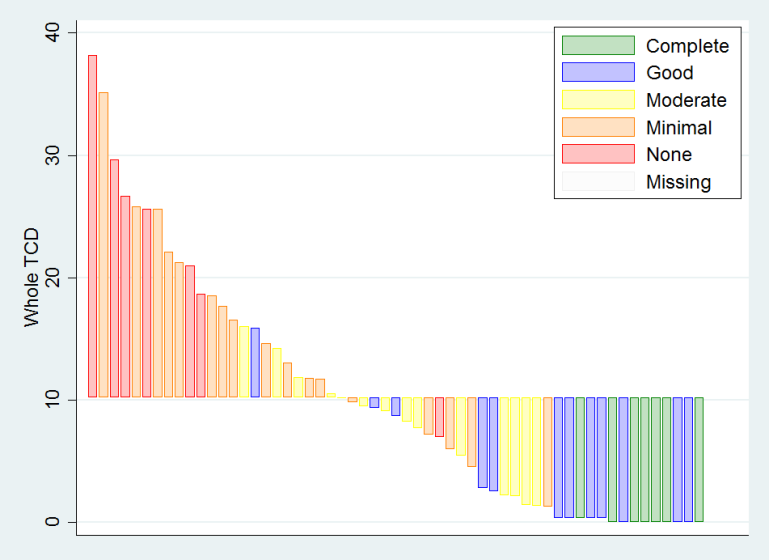** | 1. **Resection whole as percentage of biopsy TCD (p<0.001)**   **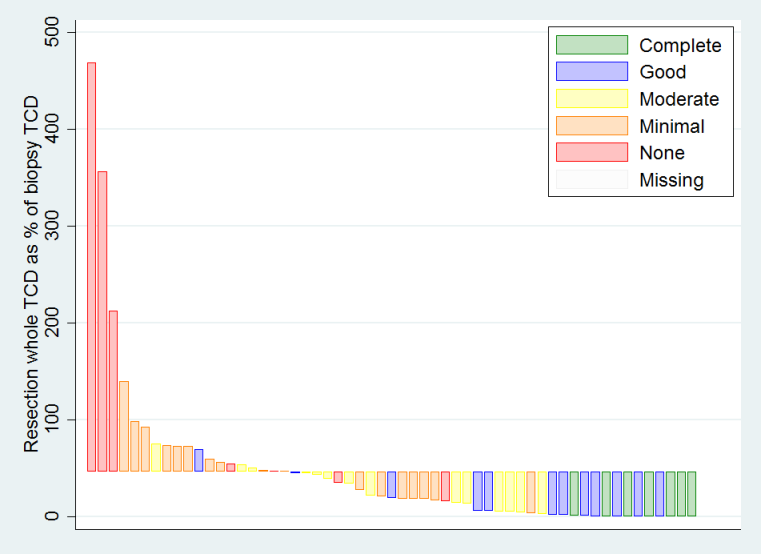** |
