# Supplementary material for: A prospective phase II study of pre-operative chemotherapy then short-course radiotherapy for high risk rectal cancer: COPERNICUS
Source: Br J Cancer. 2018 Aug 17;119(6):697–706. doi: 10.1038/s41416-018-0209-4 (PMC6173784; doi:10.1038/s41416-018-0209-4)
Supplement: Supplementary file 2 — Supplementary Table 1 [file 41416_2018_209_MOESM2_ESM.docx]

**Supplementary Table 1 (online only). Post-surgery complications within 30 days of operation**

|  |  | **n (%)*** |
| --- | --- | --- |
| Anastomotic dehiscence | Yes | 3 (5) |
|  | No | 50 (88) |
|  | Unknown | 4 (7) |
| Perineal wound dehiscence | Yes | 3 (5) |
|  | No | 50 (88) |
|  | Unknown | 4 (7) |
| Pelvic infection/collection requiring draining | Yes | 10 (18) |
|  | No | 43 (75) |
|  | Unknown | 4 (7) |
| Serious infection elsewhere | Yes | 9 (16) |
|  | No | 44 (77) |
|  | Unknown | 4 (7) |
| Serious infection elsewhere - location | Wound | 6 (67) |
|  | Spleen | 1 (11) |
|  | Unknown | 2 (22) |
| Venous thromboembolic event | Yes | 0 (0) |
|  | No | 53 (93) |
|  | Unknown | 4 (7) |
| Death within 30 days | No | 57 (100) |
| Myocardial infarction | Yes | 0 (0) |
|  | No | 53 (93) |
|  | Unknown | 4 (7) |
| Cerebrovascular accident | Yes | 0 (0) |
|  | No | 53 (93) |
|  | Unknown | 4 (7) |
| Other complications | Fast atrial fibrillation | 1 (2) |
|  | Post-operative ileus | 1 (2) |
|  | Ureteric Stenosis from pelvic collection | 1 (2) |
|  | Fluid discharge and foecal matter passing PR grade 1 | 1 (2) |
|  | Diarrhoea grade 3 | 1 (2) |
|  | Abdominal ascites grade 3 | 1 (2) |
| Second operation required | Yes | 4 (7) |
|  | No | 49 (86) |
|  | Unknown | 4 (7) |
| If yes, what was done? | Laparotomy and Hartmann's | 1 (2) |
|  | Laparotomy, Resection of loop jejunostomy & re-formation of end ileostomy | 1 (2) |
|  | Ureteric Stent | 1 (2) |
|  | Rectal suture repair of anastomosis ileus 1 week post-op | 1 (2) |
| Time spent on ITU/HDU post op (days) | 0 | 31 (54) |
|  | 1 to 3 | 17 (30) |
|  | 5 to 7 | 4 (7) |
|  | 16 | 1 (2) |
|  | Unknown | 4 (7) |
| Was patient discharged within 30 days? | Yes | 49 (86) |
|  | No | 4 (7) |
|  | Unknown | 4 (7) |
| If discharged within 30 days, time from surgery to discharge (days) | Median (IQR,range,n) | 7 (6-11,2-23,49) |
| If discharged within 30 days, re-admission necessary after discharge | Yes | 7 (14) |
|  | No | 41 (84) |
|  | Unknown | 1 (2) |
| If discharged within 30 days, urinary catheter at time of discharge? | Yes | 11 (22) |
|  | No | 38 (78) |
|  | Unknown | 0 (0) |

*unless otherwise indicated, denominator N=57 i.e. those who had surgery
